# Psychiatric co-morbidity associated with pheniramine abuse and dependence

**DOI:** 10.4103/0019-5545.46079

**Published:** 2005

**Authors:** Hemraj Pal, Rajesh Kumar, Shashi Bhushan, Neeraj Berry

**Affiliations:** * Associate Professor National Drug Dependence Treatment Centre, All India Institute of Medical Sciences, New Delhi 110029; ** Ex-Senior Resident National Drug Dependence Treatment Centre, All India Institute of Medical Sciences, New Delhi 110029; *** Ex-Junior Resident National Drug Dependence Treatment Centre, All India Institute of Medical Sciences, New Delhi 110029; **** Ex-Senior Resident National Drug Dependence Treatment Centre, All India Institute of Medical Sciences, New Delhi 110029

**Keywords:** Co-morbidity, psychotic syndromes, pheniramine abuse/dependence

## Abstract

The abuse of cough syrups containing antihistamines and codeine is being increasingly noted. The abuse of antihistamines alone has also been reported. The use of antihistamines alone or in combination with other substances of abuse may predispose individuals to develop psychiatric symptoms or syndromes as a part of intoxication, withdrawal or as co-morbid conditions. We present two case reports to highlight the occurrence of co-morbid psychopathology in association with antihistamine abuse and dependence.

Case I used high doses of pheniramine for about 2 years and became suspicious of his wife; he even doubted the paternity of his yet-to-be-born child. The associated behavioural abnormalities suggested that he was acting out on the delusion. He also had seizures associated with the intake of a high dose of pheniramine. Case II had multiple substance use, and dependence on alcohol and pheniramine. He demonstrated abnormal behaviour suggestive of psychosis and organic brain syndrome that persisted for a few days and remitted on discontinuation of the substances. These two cases demonstrate the occurrence of psychotic syndromes associated with heavy pheniramine use. The psychopathology can vary from an independent psychotic syndrome to an organic brain syndrome-like disorder.

## INTRODUCTION

Antihistamine-containing cough syrups and cough suppressants have been abused for a long time in various parts of the world,^1^ including India.^2^ Psychological tolerance and symptoms of physical withdrawal following massive diphenhydramine abuse have been documented.^3^

Brompheniramine-induced withdrawal has been reported and is characterized by nausea, tremor, generalized sweating and depression.^4^ However, pheniramine dependence has not been reported so far, although its abuse has been reported.^5^ Use/abuse/dependence of antihistamines or antihistamine-containing cough syrups is known to be associated with psychiatric syndromes and disorders.^6^

Two case reports of toxic psychosis induced by diphenhydramine and prophenpyridamine, respectively, have been reported.^7^ In one study, about 44% (*n*=43) of pheniramine abusing patients developed psychosis/delirium while only 32% (*n*=75) of patients using other antihistamines developed psychosis/delirium.^5^

The two case reports presented here illustrate the occurrence of psychotic symptoms/disorders with massive pheniramine abuse/dependence.

## CASE I

A 23-year-old married man, with secondary-level education, employed in a jewellery shop had no past or family history of substance abuse. He had a well-adjusted premorbid personality and no psychiatric or medical illness. He presented with complaints of use of pheniramine tablets for 2 years. The patient began with 2 tablets of 50 mg, gradually increased the dose to 10 tablets over a period of 15 months till about 7 months ago, when he got married. The reason he gave for taking an increased number of tablets was persistent desire, lethargy and a kind of ‘emptiness’ which would disappear after consuming the tablets. He would get ‘a kind of satisfaction’ which he could not describe in words. As he increased the intake to 10 tablets, the patient began experiencing dryness of the mouth, restlessness and difficulty in sleeping.

Since his marriage 7 months ago, he had increased the number of tablets of pheniramine to 15–20 tablets each time every second or third day. A week before contact with treatment, he consumed 27 tablets of pheniramine and had two episodes of generalized tonic–clonic seizures at an interval of 2 hours, regaining consciousness in between the seizures.

General physical examination and investigations such as routine haemogram, chest X-ray, blood sugar, liver function tests, kidney function tests, CT scan of the head were within normal limits. An electroencephalogram (EEG) done in the postictal phase showed epileptic discharge.

For the past 7 months, he had been suspicious of his wife. Suddenly, he was struck by the thought that she had sexual relations with someone and became convinced about this. The patient would pressurize his wife to reveal the name of the person. He further intensified efforts to get his wife to reveal the name of the person with whom she was having a relationship. Gradually, the family members, particularly his father, also got implicated in the belief. The patient moved to a different floor in the same house away from his family.

During the mental state examination, he enquired several times about any test that would prove the paternity of the expected child. The mental state examination revealed normal psychomotor activity, normal speech, euthymic affect, delusion of infidelity, and no perceptual abnormality with poor insight. His belief remained as such over the following month.

## CASE II

A 28-year-old man, educated till class twelve, with no past or family history of psychiatric illness or substance depen-dence/abuse, presented with complaints of alcohol use for 15 years, pheniramine use for the past 5 years and increased irritability, suicidal ideation and sadness of mood for the past 6 months. The patient began consuming alcohol 15 years ago and gradually increased the amount. Since the past 1 year, he would also drink in the morning and experience physical withdrawal symptoms in the form of tremor, anxiety, light-headedness when he would not get alcohol. About 5 years ago, the patient was introduced to pheniramine by a friend as he was unable to afford alcohol and needed some cheaper alternative for a ‘high’. He started with 10 tablets of 25 mg daily in two divided doses. Soon the dose escalated to 20 tablets and over the next 3 years, to 30 tablets of 25 mg each. The patient felt that his experiences could be described as being similar to that with cannabis (*bhang*), as he could differentiate it from the experiences of alcohol or tobacco intake.

The characteristic experiences described by the patient were a sense of euphoria, memory lapses, decreased awareness of the surroundings, persistence of visual scenes beyond the time that they had existed, e.g. after watching television, despite the scene having ended he would feel as if the scene had been pasted on his mind. He also described symptoms suggestive of motor incoordination, slurring of speech, ataxia, generalized weakness and lack of strength in the limbs.

The patient gave a history of two episodes suggestive of alcohol withdrawal seizures as well. On the first occasion, the patient had cut down his usual amount of alcohol intake due to high-grade fever and loose motions. He developed altered consciousness along with tonic–clonic movements; the entire episode lasted for about 5 minutes followed by postictal confusion for the next half-an-hour. A similar episode occurred about a month before admission.

The patient complained of increased irritability, sadness of mood, suicidal ideation, decreased interest in work and the surroundings, and decreased sleep and appetite. Physical examination and routine investigations revealed no abnormalities except for hepatomegaly. The mental state examination showed depressed cognition with no abnormalities of speech or perception. He was oriented to time, place and person and had no impairment in judgement or abstraction.

In the ward, the patient had intense craving for pheniramine and alcohol, and coarse tremors and anxiety, which subsided over the next 10 days. Psychological testing for personality revealed cluster B traits and depressive features. The patient was started on fluoxetine 20 mg per day, which was increased to 40 mg per day before discharge, and motivation enhancement sessions were planned.

## DISCUSSION

Abuse of antihistamine-containing cough syrups and cough suppressants has been reported from various parts of world, including India.^1^,^2^ Though chlorpheniramine is one of the commonest antihistamines to be abused, dependence has not been reported so far.

Case I had dependence on chlorpheniramine and also developed psychosis characterized by a delusion of infidelity, which is in keeping with the literature. The patient also had other documented experiences including seizures after taking more than 20 tablets of 50 mg chlorpheniramine indicating overdose phenomena. The patient in Case II had a background of alcohol dependence along with heavy pheniramine use, which led to unusual experiences after taking chlorpheniramine. Along with dependence, this patient had abnormal and vivid experiences after taking chlorpheniramine, probably as a part of intoxication. Depressive features, which could be a part of cluster B traits, particularly borderline traits, were also present.

To conclude, it can be said that antihistamines, particularly chlorpheniramine, are associated with the development of psychotic phenomenology, especially in predisposed indivi-duals. Thus it is important to evaluate for psychosis in patients who have abused or have dependence on antihistamines.
